# Effect of selenium and methods of protein extraction on the proteomic profile of *Saccharomyces* yeast

**DOI:** 10.1515/biol-2022-0496

**Published:** 2022-09-09

**Authors:** Marek Kieliszek, Adam Waśko, Katarzyna Michalak, Anna M. Kot, Kamil Piwowarek, Stanisław Winiarczyk

**Affiliations:** Department of Food Biotechnology and Microbiology, Institute of Food Sciences, Warsaw University of Life Sciences – SGGW, Nowoursynowska 159 C, 02-776 Warsaw, Poland; Department of Biotechnology, Microbiology, and Human Nutrition, University of Life Sciences in Lublin, Skromna 8, 20-704 Lublin, Poland; Department of Epizootiology and Clinic of Infectious Diseases, Faculty of Veterinary Medicine, University of Life Sciences in Lublin, Głęboka 30, 20-612 Lublin, Poland

**Keywords:** selenium, *Saccharomyces cerevisiae*, yeast, proteomics, MALDI TOF/TOF

## Abstract

Selenium may influence the biosynthesis of individual proteins in the yeast cell cytosol. In this study, we used two-dimensional (2D) electrophoresis to identify proteins that are differentially expressed by the enrichment of selenium in *Saccharomyces cerevisiae* yeast cells. We chose eight protein fractions for further proteomic analysis. A detailed analysis was performed using the Ultraflextreme matrix-assisted laser desorption/ionisation time-of-flight/time-of-flight mass spectrometer, which enables fast and accurate measurement of the molecular weight of the analysed proteins. This study, for the first time, provides evidence that selenium-enriched yeast contains higher levels of mitochondria malate dehydrogenase, adenosine-5′-triphosphate (ATP)-dependent RNA helicase dbp3, and tryptophan dimethylallyltransferase, and alanyl-tRNA editing protein AlaX than yeast without the addition of selenium. It should be emphasised that the proteomic variability obtained reflects the high biological and complexity of yeast metabolism under control and selenium-enriched conditions and can be properly used in the future as a model for further research aimed at determining the expression of appropriate metabolic genes.

## 1 Introduction

For many organisms, selenium (Se) is a trace element necessary for life [1]. First of all, selenium helps to maintain immune endocrine and metabolic and cellular homeostasis [2]. Its key role is to defend cells against oxidative stress. This element, being a component of many selenoproteins with biological functions, can act as a cofactor of various enzymes, including glutathione peroxidase [3]. By participating in antioxidant defence systems, the appropriate dose and chemical form of selenium play a preventive role against the formation of free radicals and reactive oxygen species (ROS). Thus, it may prevent the development of many diseases in higher organisms [4]. The main reason yeast is used as a source of this element is that it is a model eukaryotic organism. Genetic and biochemical studies on the species *Saccharomyces cerevisiae* allowed us to understand the many basic processes taking place in eukaryotic cells of higher organisms [5]. The simplicity of breeding, easy phenotypic testing, and genetic manipulation presents a full-scale comparison of the human body. Due to the fact that the effect of selenium on the human body is not fully known, studies are being carried out confirming the effect of the bioelement on yeast, in particular *S. cerevisiae*. Yeast cells are very interesting biological materials used in many studies. In addition, *S. cerevisiae* yeast is one of the basic model eukaryotic organisms. Moreover, its rapid growth, dispersed cells, and ease of replication on plates made it possible to broaden the knowledge of these microorganisms. Therefore, it is of great importance to conduct research that significantly broadens the knowledge of modern molecular biology of eukaryotic cells.

Ionic forms of selenium found in the natural environment can undergo metabolic processes in yeast cells. Single-celled microorganisms are able to incorporate elements into their cellular structures. Due to the presence of organic and inorganic forms of selenium in the environment, yeast has adapted to bind both forms [6,7]. The most important factors responsible for the bioavailability of selenium for yeast include the active acidity (pH) of the environment and the level of oxygen dissolved in the culture medium and temperature. It was noticed that an increase in pH slows down the accumulation of the element in cells [8]. In the case of selenium accumulation inside the cell, active specialised transport is the main basis. However, it should be mentioned that the mechanism of action of the molecular carriers that transport selenium is not well understood. It can be assumed that sulphate permease (Sulp) is responsible for the translocation of an element in yeast, and the high similarity to sulphur allowed the use of the Sul1p and Sul2p transport systems for the transport of SeO_3_^−2^ to the cell cytosol [9]. It is worth noting that the appropriate form and dose of selenium can be a stress factor for yeast cells. Therefore, some species of yeast have developed detoxification mechanisms allowing them to increase tolerance to various stress factors (including elements). Apart from morphological changes in cells, the metabolic changes in the cellular cytosol also influence an increase in selenium tolerance. The presented phenomena may be a factor that will lead to changes in the permeability of membrane channels, changes in the activity of relevant enzymes, or changes in the expression of specific genes in yeast. Proper transport of substances to and from the cell and between organelles is essential for the proper functioning of cells. This transport is carried out mainly through endocytic vesicles made of plasma membranes. Therefore, cells have developed numerous methods of adaptation to unfavourable environmental conditions, such as the accumulation of spare sugars or detoxification processes to break down or assimilate unfavourable components [10]. The fact that yeast is able to accumulate significant amounts of selenium inside the cell means that it can be used in the future as a functional additive aimed at enriching food with this highly functional microelement, thus reducing the risk of deficiency of this element in the diet.

It is worth noting that the protein content and expression level can have a great influence on the condition and changes in the life cycle of yeast cells. Furthermore, they can be correlated with the variability of the species and a given yeast strain. Identifying and studying the metabolic functions of yeast by conventional methods is often difficult, time-consuming, and sometimes ambiguous. Therefore, the use of proteomics research can be an effective approach in many scientific fields. Two-dimensional gel electrophoresis (2-DE) is able to visualise an image of protein fractions [11], which reflects changes in the level of protein abundance, isoforms, or various post-translational modifications, and enables the separation of complex mixtures of proteins according to the isoelectric point and molecular weight. Therefore, in this article, we used a combination of these methods to identify proteins and obtain a comprehensive picture of selected proteins found in yeast cells. The results obtained in this way may provide an answer as to how stressful conditions resulting from the presence of selenium in the culture medium can influence the expression of proteins. We still do not know the operation of all mechanisms responsible for the regulation and control of specific changes in protein expression that may be induced as a result of stress. To achieve these goals, it is essential to understand the physiological and metabolic mechanisms used by yeast cells to obtain adequate morphological protection. This is to limit damage and withstand many unfavourable breeding conditions. It should be emphasised that, currently, great importance is attached to the search for protein indicators of yeast tolerance to stress (including the presence of selenium). Thus, thanks to proteomic methods, we can observe many proteins at the same time and show their importance and the modifications caused by various stress factors that interfere with the growth and development of yeast cells.

Extensive proteomic studies of yeast cells in response to selenium stress have not been performed so far. In this study, we used 2-DE and the MALDI TOF/TOF method to evaluate the emergence of selected proteins in the biomass of *S. cerevisiae* ATCC MYA-2200, the expression of which changes in response to high levels of stress related to the presence of selenium in the culture medium.

## 2 Materials and methods

### 2.1 Biological material and microbiological media

The research used the *Saccharomyces cerevisiae* ATCC MYA-2200 yeast strain from the pure cultures collection of the Department of Biotechnology and Food Microbiology at Warsaw University of Life Sciences-SGGW.

For the purpose of cultivation, YPD liquid medium of a standard composition (g/L) – glucose (dextrose) 20, peptone 20, and yeast extract 10 was used. YPD liquid medium enriched with sodium selenite(iv) (Sigma-Aldrich, Poland) was used as the experimental medium for submerged cultivation. The pH of the media was maintained at 5.0. Media as well as aqueous solutions of Na_2_SeO_3_ salts were sterilised at 121°C for 20 min (Hirayama Autoclave HG80, Japan). Then, the saline working solution was added to the sterile YPD media in such volumes that the final selenium content of the experimental media was 20 mg Se^4+^/L.

### 2.2 Yeast cultivation

For the experimental purpose, cultures were grown in 0.5 L flasks containing 90 mL of liquid (control) or experimental medium. Medium was inoculated with a 10% volume of cell suspension propagated in inoculation culture (6.0 × 10^8^ cfu/mL). A cellular biomass of *S. cerevisiae* yeast strains was obtained after shaking the culture for 24 hours (Innova 44^®^ Inc Shaker, New Brunswick™, USA) at a vibration amplitude of 200 cycles/min in the control YPD liquid medium and experimental YPD enriched with selenium. Three independent replications of the cultures were carried out. After cultivation, the obtained yeast biomass was centrifuged (3,000×*g*, 10 min, +4°C, Centrifuge 5804 R Eppendorf, Germany) and then rinsed twice with sterile distilled water. The biomass obtained was freeze-dried and stored for further analysis.

### 2.3 Protein extraction

Lyophilised yeast samples (0.05 g) were subjected to protein extraction by various methods: 15 min 50 mM TRIS extraction in an ultrasonic bath (A), 15 min ultrapure water (MQ water) extraction in an ultrasonic bath (B), 5 min vortexing in 50 mM TRIS with glass beads (C), 5 min vortexing in MQ water with glass beads (D), freezing–thawing of samples in 50 mM TRIS (E), and freezing–thawing of samples in MQ water (F). In each case, the volume of solvent used was 5 mL. After extraction, the solutions were centrifuged (20 min, 5,000×*g*, 4°C). The supernatants received were subjected to protein concentration measurements using the spectrophotometric method by measuring absorbance at 280 nm (MaestroNano Micro-Volume Spectrophotometer, Maestrogen, Taiwan).

### 2.4 Protein purification and precipitation

The protein mixture obtained by TRIS extraction assisted with glass beads was purified, desalted, and concentrated by Amicon Ultra-0.5 3 kDa centrifugal filter units (Merck KGaA, Darmstadt, Germany). The concentrated solution was then subjected to protein precipitation using a precipitation kit (Ready-Prep™ 2-D Cleanup Kit, Bio-Rad, Warsaw, Poland) to obtain a pellet containing 200 µg of protein. The protein precipitate was dissolved in 300 µL of rehydration buffer containing thiourea (Bio-Rad, Warsaw, Poland), transferred to a rehydration plate, and covered with 17 cm immobilised pH gradient linear strips for isoelectric focusing (pH 3–10, Bio-Rad, Warsaw, Poland) flooded with mineral oil (pH 3–10, Bio-Rad, Warsaw, Poland) and left overnight [12].

### 2.5 2D-electrophoresis

Next the soaked strips were subjected to isoelectric focusing (in the first dimension of electrophoresis) in the IEF-100 Hoefer apparatus (Hoefer IEF100, Hoefer, Inc., Holliston, MA, USA). The process conditions were as follows: 250 V/30 min; 10,000 V/3 h; 60 kV/h, with a current limit of 50 μA/strip. Before the second dimension, the focused strips were equilibrated sequentially in 1.4-dithiothreitol and iodoacetamide solutions. Each stage lasted 15 min. The equilibrated strips were subjected to molecular mass separation by vertical electrophoresis in 12.5% polyacrylamide gel placed in an electrophoretic chamber (PROTEAN^®^ II xi, Bio-Rad, Warsaw, Poland). Five repetitions of gels were performed. The current parameters were as follows: 600 V/30 mA/100 W. After separation, the gels obtained were silver stained with silver nitrate in the presence of formaldehyde and digitalised by scanning (Image Scanner III, GE Healthcare, Warsaw, Poland) [13].

### 2.6 Graphical analysis and preparation for MS identification

All gel images were processed by Delta2D software (version 4.7, DECODON, Greifswald, Germany). The images were connected by automatic warping. False-positive and false-negative spots were also manually excluded. Next all gel images were combined into one synthetic fused image, which included representatives for all spots from the project. Ratio values were determined by Delta2D after gel warping and obtaining a fused image (ratios greater than 1.3 were assigned as upregulated, whereas smaller than 0.65 were assigned as downregulated). For this purpose, a ratio of mean spot volume for the selenium group to the control group was given. In every case, the ratio was determined for two selenium spots and two control spots. The expression ratios were generated, and statistics were made over normalised volumes by *T*-test with Welch approximation (*p*-value ≤ 0.05). On this basis, spots for identification were designated, cut from gels, destained, reduced, and alkylated using dithiothreitol and iodoacetamide solutions. After this preparation, the gel pieces containing proteins were subjected to digestion to obtain peptide fragments necessary for identification. In this case, trypsin (Trypsin Gold, Promega, Madison, WI, USA) in 50 mM ammonium bicarbonate buffer was used for digestion, which occurred at 37°C for 12 h. Subsequently, peptides were eluted from gel fragments by triple extraction with a water/acetonitrile/TFA solution (v:v 45:50:5). Extracts were concentrated and purified by C18 Zip-TIP pipette tips according to the manufacturer’s instructions (PR 02358, Technical Note, Merck Chemicals, Billerica, MA, USA) [14].

### 2.7 Matrix-assisted laser desorption/ionisation-time-of-flight (MALDI-TOF) protein identification

Samples prepared in the previous step were spotted on an Anchor Chip MALDI plate (Bruker, Bremen, Germany) and covered by 1 μL of α-cyano-4-hydroxycinnamic acid matrix (HCCA, Bruker, Bremen, Germany). Simultaneously, a peptide standard solution (Peptide Calibration Standard II, Bruker, Bremen, Germany) was spotted and covered by the acid matrix on the calibration spots. Mass spectra were recorded in active, positive reflector mode within the 700–4,000 *m/z* range using an Ultraflextreme MALDI TOF/TOF (Bruker, Bremen, Germany) spectrometer and the flexControl 3.3 (Bruker, Bremen, Germany) software. The spectra collected were smoothed and baseline corrected. The peak list generated in the flexAnalysis 3.0 software (Bruker, Bremen, Germany) with a signal-to-noise ratio greater than 3 was transferred to BioTools 3.2 (Bruker, Bremen, Germany) and compared to the Mascot 2.2 software (Matrix Science, Boston, MA, USA) using the Swiss-Prot database (www.uniprot.org) restricted to “fungi” taxonomy with a maximum error of 0.3 Da and carbamidomethylation of cysteine as an obligatory modification. The results with a Mascot score above 58 for other fungi and greater than 51 for *Saccharomyces cerevisiae* were considered statistically significant (*p* ≤ 0.05); otherwise, the fragment ion spectra of chosen peptides were obtained using the LIFT mode and combined with the aim of MALDI TOF/TOF identification. Gene Ontology analysis of the differentially expressed proteins was performed using the internet software PANTHER classification system (v.14.0) (http://www.pantherdb.org/).

## 3 Results and discussion

### 3.1 Method of protein extraction from yeast biomass

Yeast cells, along with the industrial production of proteins [15], have contributed much to the development of biotechnology. It is worth noting that most proteins have an intracellular location. The use of the simplest and most cost-effective methods from the point of view of the basic process of extracting proteins from yeast cells is of key importance in studying the biological functions of these proteins. As a result of the analysis, it was found that the highest protein content (4.402 mg/mL) from yeast biomass was obtained using 5 min vortexing in 50 mM TRIS with glass beads (Table 1). The lowest protein content (1.832 mg/mL) was obtained using the extraction method in a TRIS buffer with the use of ultrasound. The effectiveness of the presented method is varied, but with much better results – resulting from the efficiency of the protein obtained – after the destruction of the cell walls. This method was used in later experiments due to its ease and efficiency. On the basis of the results obtained, it is reasonable to suppose that the liquid in the form of a TRIS buffer in combination with glass beads can be used as an efficient and durable reagent for cell lysis, enabling easy extraction and analysis of proteins. Currently, the most common methods of extracting proteins include the mechanical disruption of cells and performing extraction by chemical methods [16]. It is worth noting that there is more and more interest around the world in the use of the pulsed electric field to improve the extraction of various metabolites, including proteins, from microorganisms [17]. According to Ge et al. [16], the reagent 3-(dimethylamino)-1-propylamine formate ([DMAPA] FA) can also be used for the efficient extraction of proteins from yeast cells. Glass spheres are also an efficient and reliable method for breaking the wall-membrane assembly in yeast cells. The mechanical energy generated enables the cytoplasm content to be released quickly from yeast cells [18]. The prospects for the use of yeast biomass resulting from their ability to quickly grow on various culture substrates and the possibility of obtaining a high content of valuable protein make it an excellent organism for research on the influence of selenium on proteomic changes in the cellular cytosol.

**Table 1:** Protein extraction from *Saccharomyces cerevisiae* ATCC MYA-2200

Extraction method	Protein concentration (mg/mL)
15 min 50 mM TRIS extraction in ultrasonic bath (A)	1.832 ± 0.006
15 min ultrapure water (MQ water) extraction in ultrasonic bath (B)	3.003 ± 0.026
5 min vortexing in 50 mM TRIS with glass beads (C)	4.402 ± 0.058
5 min vortexing in MQ water with glass beads (D)	4.022 ± 0.029
Freezing–thawing of samples in 50 mM TRIS (E)	2.728 ± 0.059
Freezing–thawing of samples in MQ water (F)	2.403 ± 0.017

### 3.2 Profile 2-DE

**Figure 1:**
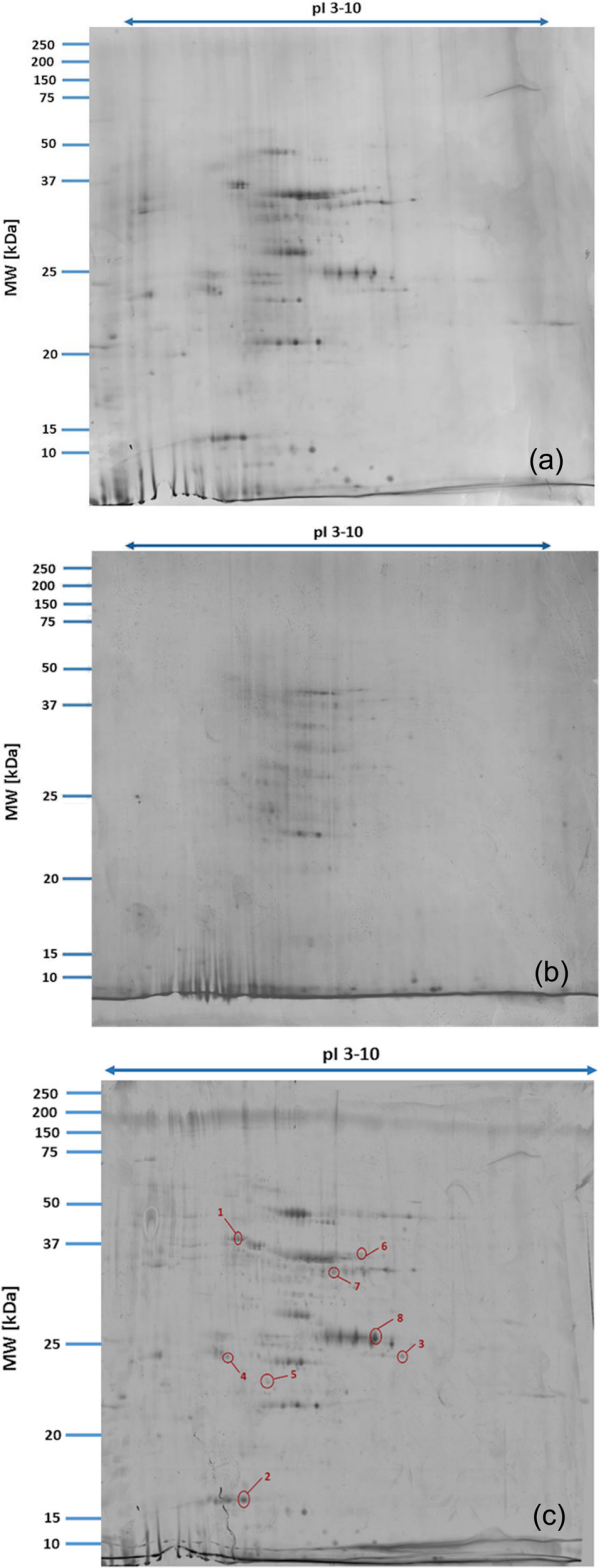
Image of a 2D PAGE experiment demonstrating electrophoretic separation of proteins extracted from *S. cerevisiae* ATCC MYA-2200 cells cultured in the absence (a), presence of 20 mg Se^4+^/L (b), fused image showing protein spot patterns from the experiment (c). Linear wide range gradients of immobilised pH (pH 3–10) in the first dimension and on 12% SDS-PAGE for the second dimension. The spots marked with small arrows indicate proteins with altered expression under the influence of selenium.

**Figure 2:**
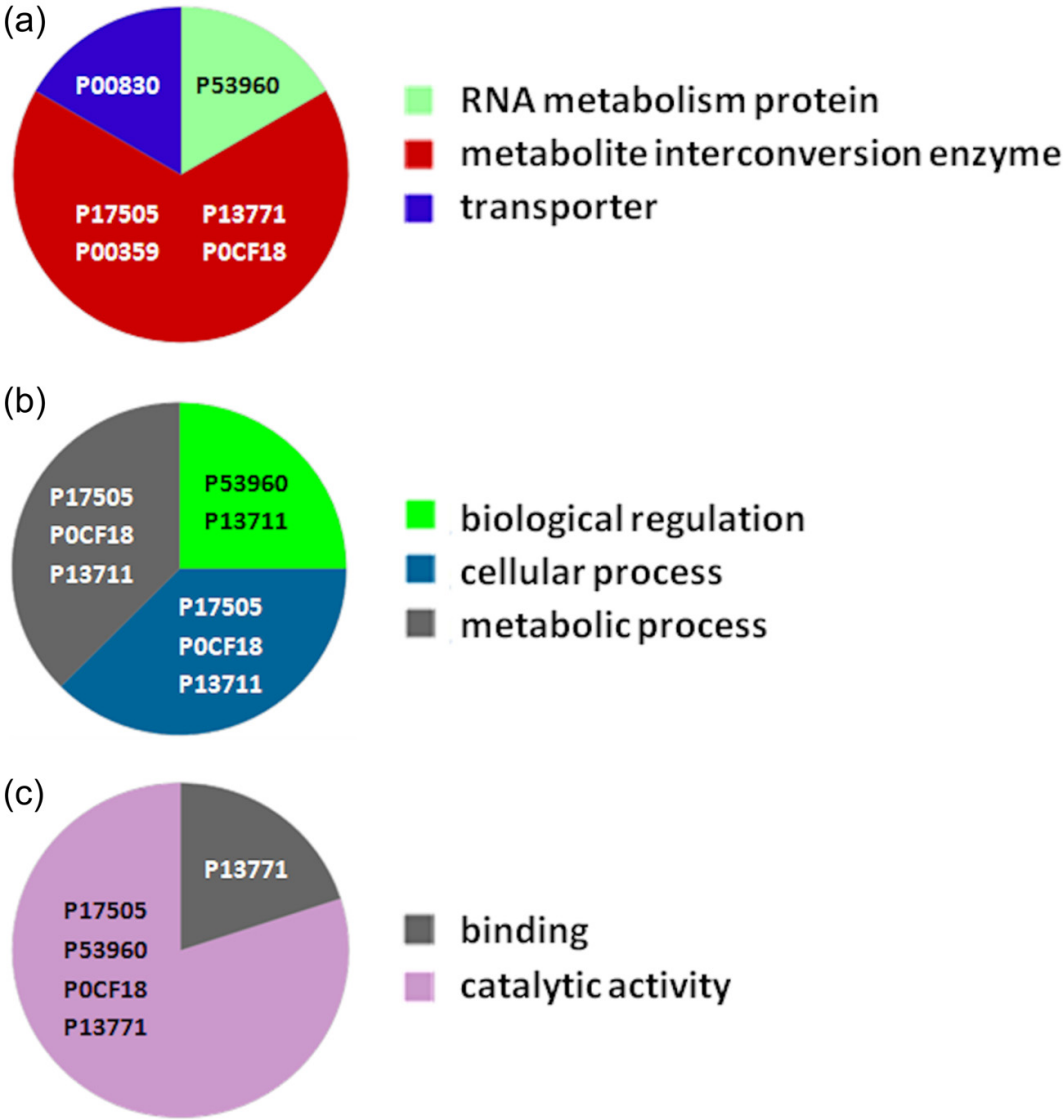
Gene Ontology analysis of the differentially expressed proteins; protein class (a), biological process (b); and molecular function (c).

Proteomic analyses carried out on various biological matrices using the latest identification methods are an excellent tool used in many biotechnological fields. As a result of the research, it was found that the synthesis levels of the protein spots obtained are influenced by the presence of selenium (Figure 1). Proteins showing reduced synthesis caused by the influence of this element in the culture environment on the *S. cerevisiae* strain are the enzyme Acyl-CoA oxidase, which is involved in the peroxisomal β-oxidation system, the beta subunit of ATP (mitochondrial) synthase and glyceraldehyde-3-phosphate dehydrogenase (GAPDH) (Tables 2 and 3). As a result of the gene ontology analysis of differentially expressed proteins, we showed that Acyl-CoA oxidase, malate dehydrogenase (MDH) (mitochondrial), and uncharacterised protein YMR085W are involved in metabolic processes in yeast cells. In the case of the ATP synthase, subunit beta protein (mitochondrial) regulates the flow of various substances in the cell cytosol in a controlled manner (class of transmembrane proteins). The molecular and biological functions, as well as protein classifications, are presented in Figure 2. It is worth emphasizing that in response to various environmental stresses, a common strategy adopted by cells is to increase the expression of stress-protective genes and to reduce protein synthesis and the activity of growth-related genes [19]. According to the literature data, the first enzyme (Acyl-CoA oxidase) plays a key energetic function in the processes of fatty acid conversion [20]. The lack of a properly functioning Acyl-CoA oxidase inhibits four successive reactions: dehydrogenation, hydration, dehydrogenation, and the thiolytic cleavage of shorter fatty acids (FAs) and long-chain fatty acids (VLCFAs) [21]. At the same time, damage to the peroxisomal β-oxidation process increases the concentration of VLCFAs in the cellular cytosol [22]. The result of the processes taking place is the possibility of the formation of mutations. Selenium stress may increase the occurrence of protein misfolding and cause the loss of the function of key factors in carbon metabolism and protein synthesis pathways. Furthermore, the presence of selenium causes changes in the morphological characteristics of cells, which has been documented in many scientific publications [23,24,25,26]. The physical and chemical properties of the cell membrane are very important for the smooth functioning of the cell. These include, but are not limited to, the thickness, the flowability/stiffness of the diaphragm, and the curvature. We suggest that a consequence of damaging the activity of the peroxisomal β-oxidation process is a change in the composition of the wall structure and the membrane complex (in order to maintain its proper function between the cell and its surroundings [i.e., the presence of selenium]). Such effects greatly influence cell membrane depolarisation, which results from a decrease in the electric potential difference between the cytosol and the culture environment. Owing to the higher content of phospholipids and saturated FAs as well as stronger hydrophobic interactions, the fluidity of the membrane decreases [1]. Increasing the presence of VLCFAs with unique properties affects the stretching of both layers of the lipid membrane, potentially contributing to its stabilisation [27]. Furthermore, these properties largely determine the composition of the cell membrane, in which we can distinguish mainly phospholipids and sterols [28]. According to Alexandre et al. [29], the increase in cell membrane fluidity may be correlated with a decrease in the sterol:phospholipid and sterol:protein ratios. It is worth noting that the acyl groups of phospholipids may differ in both length and degree of unsaturation, which makes them critical determinants of the stress conditions [27]. In response to the stress caused by the presence of selenium, cells may try to maintain the appropriate physicochemical properties of the membrane by changing its composition. We used a dose of 20 mg Se^4+^/L because, in our previous publications [1], this dose may be recommended for the production of selenium amino acids in the cellular biomass of *Saccharomyces cerevisiae* (selenomethionine [SeMet] and selenocysteine [SeCys]).

**Table 2:** Representatives of 2-DE gel spots in response to selenium stress in the *S. cerevisiae* ATCC MYA-2200 yeast using mass spectrometry

ID	Protein	Accession number (UniProtKB)	Organism	Score	Match	MW (Da)	pI	Modif.	Seq. Cov (%)	Rt*	Peptide mass tolerance [Da]
1	ATP synthase subunit beta, mitochondrial	P00830	*Saccharomyces cerevisiae* (strain ATCC 204508/S288c)	152	15	54,817	5.52	C, O	29	0.108	0.06
2	Uncharacterised protein YMR085W	P0CF18	*Saccharomyces cerevisiae* (strain ATCC 204508/S288c)	89	12	49,269	6.11	C, A	33	0.330	0.17
3	MDH, mitochondrial	P17505	*Saccharomyces cerevisiae* (strain ATCC 204508/S288c)	65	9	35,685	8.46	C, O	20	3.075	0.15
4	ATP-dependent RNA helicase dbp3	A1C5V3	*Aspergillus clavatus* (strain ATCC 1007/CBS 513.65/DSM 816/NCTC 3887/NRRL 1)	58	14	54,889	9.25	C, O	33	1.407	0.17
5	Acyl-coenzyme A oxidase	P13711	*Saccharomyces cerevisiae* (strain ATCC 204508/S288c)	117	10	84,445	8.64	C, O	15	0.174	0.28
6	Trp-DMAT 1	P0CT20	*Claviceps purpurea*	63	10	51,925	5.64	C, O	27	1.253	0.21
7	Putative alanyl-tRNA editing protein AlaX	P53960	*Saccharomyces cerevisiae* (strain ATCC 204508/S288c)	55	14	50,964	6.13	C, O	37	1.980	0.20
8	GAPDH 3	P00359	*Saccharomyces cerevisiae* (strain ATCC 204508/S288c)	108	19	35,838	6.46	C, O	55	0.324	0.21

Abbreviations: C – carbamidomethylation of cysteine; O – oxidation of methionine; A – acetylation of protein N-term; Listed molecular weights (MW) and pI values correspond to the MASCOT search result, *Rt (Ratio) quotient of the group means of relative spot volumes; volume of a given spot in control group is the denominator of the ratio parameter. MW – molecular weight; pI – isoelectric point.

**Table 3:** Differentially expressed proteins in response to selenium stress in the *S. cerevisiae* ATCC MYA-2200

Protein spot	Protein name	Fragment	
		Control (mg Se^4+^/L)	20 mg Se^4+^ /L
1	ATP synthase subunit beta, mitochondrial	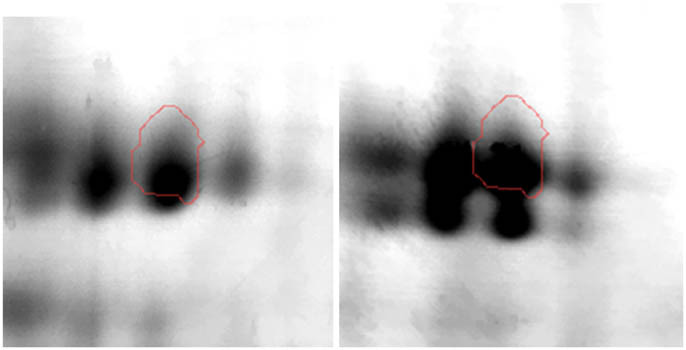	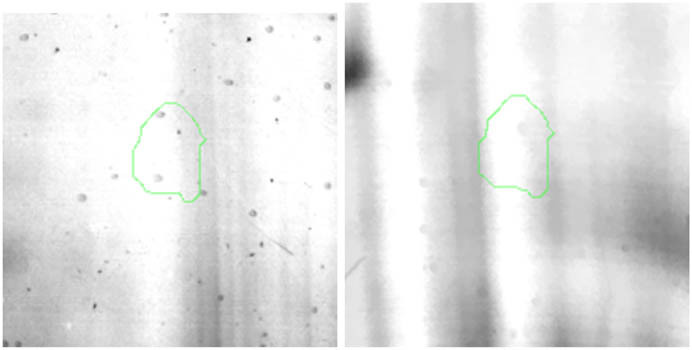
2	Uncharacterised protein YMR085W	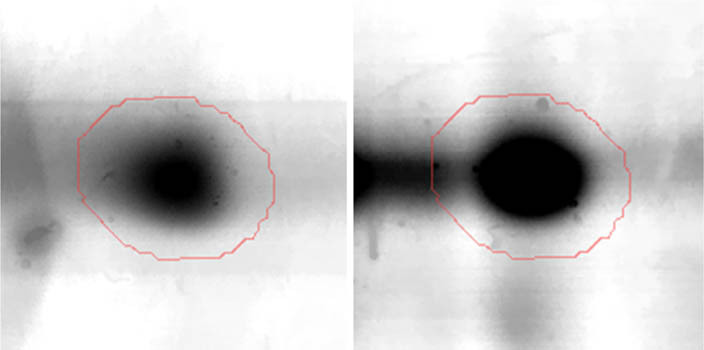	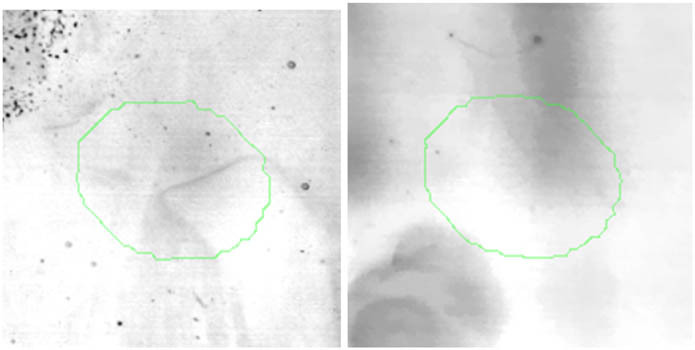
3	MDH, mitochondrial	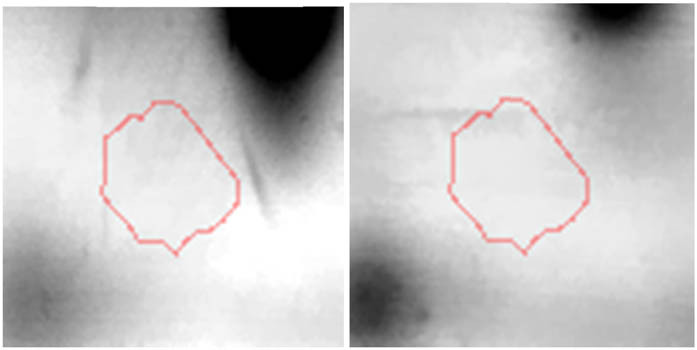	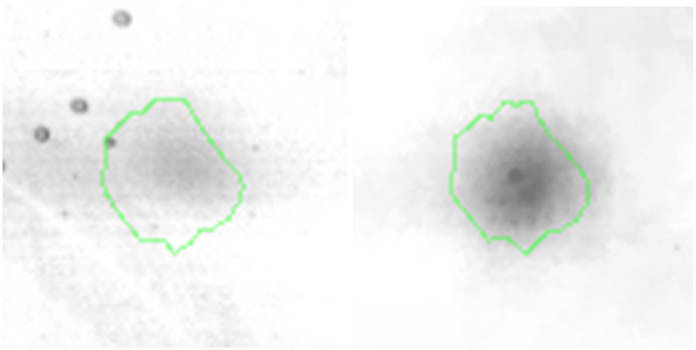
4	ATP-dependent RNA helicase dbp3	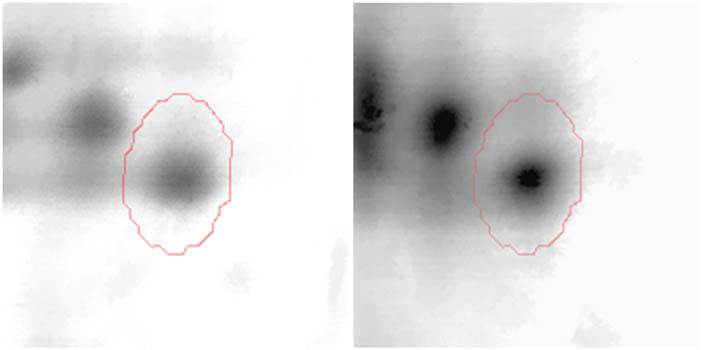	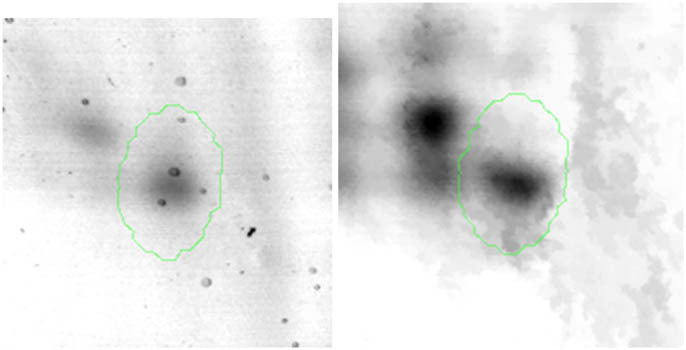
5	Acyl-coenzyme A oxidase	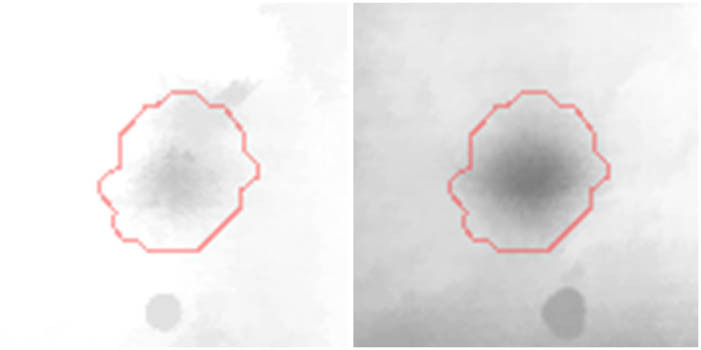	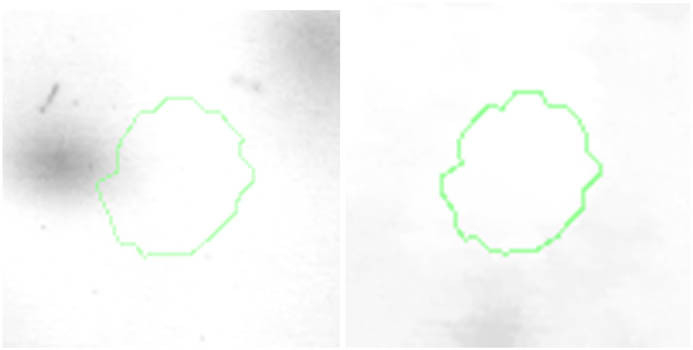
6	Trp-DMAT 1	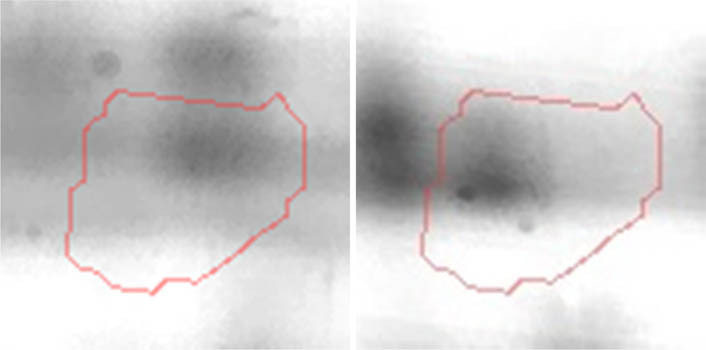	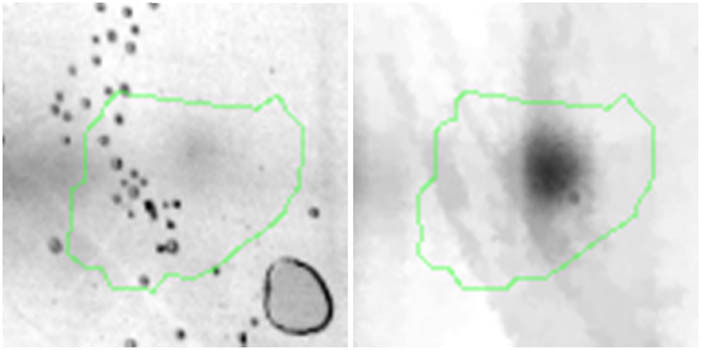
7	Putative alanyl-tRNA editing protein AlaX	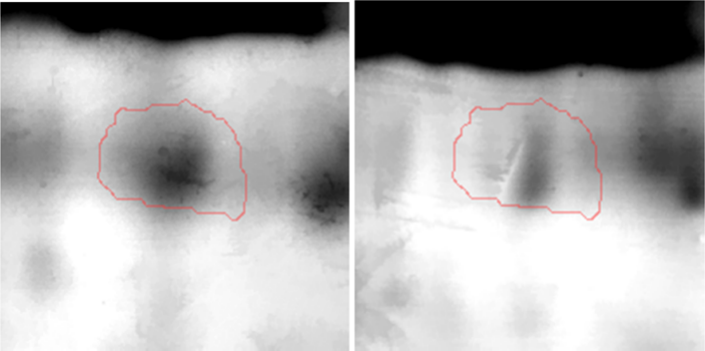	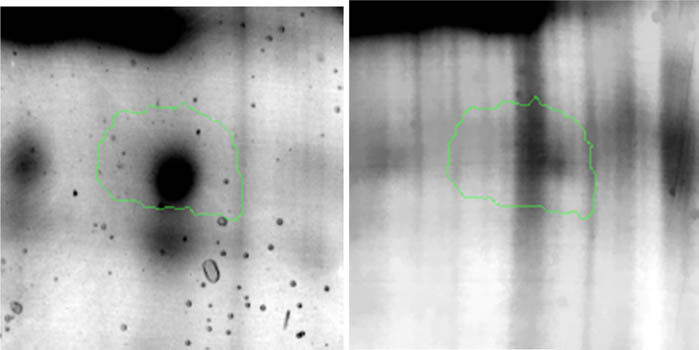
8	GAPDH	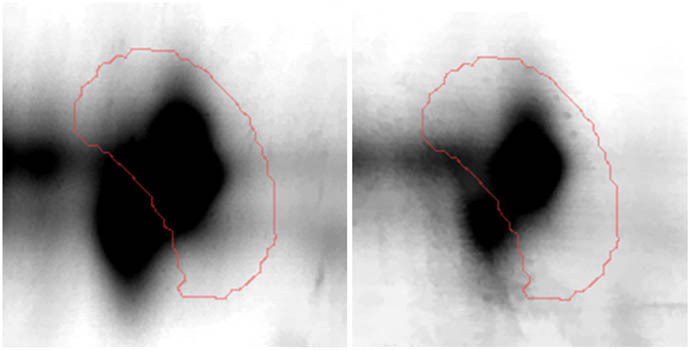	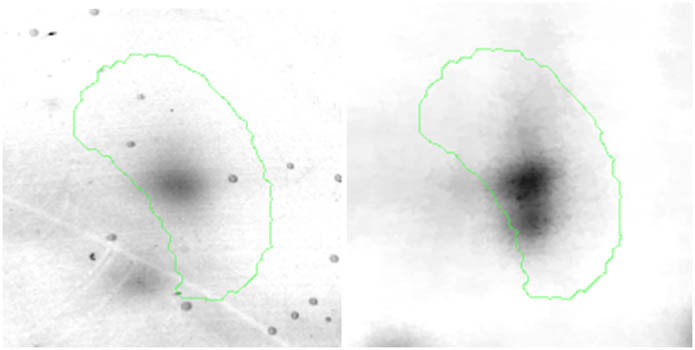

In the process of glycolysis, most genes are controlled by a series of interconnected multi-step processes. The study showed a decrease in the abundance of the GAPDH enzyme in *S. cerevisiae* ATCC MYA-2200 yeast cells. This observation suggests that selenium has a negative effect on the expression of glycolytic genes. In *S. cerevisiae* yeast cells, GAPDH is a homotetramer composed of three isoforms encoded by TDH1, TDH2, and TDH3 [30]. According to Wang et al. [31], GAPDH is a protein with many known functions, including transcriptional activation, signal transduction, cellular apoptosis, and cellular stress response. One of the most important metabolites formed in the process of glycolysis is glyceraldehyde 3-phosphate. Inhibition of GAPDH synthesis may result in the appearance of glycated proteins involved in the development of pathological processes in cells [32]. Thus, the impairment of metabolism in yeast caused by a functional change in this protein under the influence of selenium stress may be associated with a decreased survival of yeast cells.

The most common stress faced by a yeast cell is oxidative stress because the production of ATP through endogenous oxidative phosphorylation pathways causes the formation of ROS. Long-term or acute exposure to ROS may cause both reversible and irreversible damage to DNA, RNA, proteins, and lipids in cells [33]. The influence of selenium diametrically decreased the abundance of the second enzyme, beta ATP synthase (mitochondrial). This enzyme catalyses the production of ATP from adenosine-5′-diphosphate (ADP) and inorganic phosphate, and thus it is responsible for the participation in key processes of energy production by yeast cells [34]. The influence of selenium could cause oxidative damage to mitochondrial proteins, leading to a collapse of the mitochondrial membrane potential. The inhibition of ATP synthase by selenium reduces the viability of yeast cells. The research results obtained were referred in other publications, where it was found that the presence of selenium in the culture medium reduces the number of microorganisms [35,36]. A decrease in the production of ATP in the mitochondria can reduce the efficiency of energy-dependent processes such as the defence of yeast cells against the environmental stress caused by the presence of selenium.

The statistical analysis performed, characterizing protein profiles, allowed for the identification of individual groups of proteins for which the influence of selenium in the experimental medium on yeast cells was crucial in the changes in protein abundance. The research results obtained may be of great prognostic importance in further analyses aimed at determining the influence of selenium on the functioning of yeast cell metabolism.

In this study, the results obtained showed that the dynamic changes in the proteome in response to the conditions of selenium in the experimental medium are directly related to the transcription, translation, and metabolism in yeast cells. In the yeast *S. cerevisiae* ATCC MYA-2200 obtained after cultivation in a medium with the addition of selenium, an excessive abundance of the following proteins was found: mitochondria MDH, ATP-dependent RNA helicase dbp3, tryptophan dimethylallyltransferase (Trp-DMAT), and proteins that can be alanyl-tRNA editing protein AlaX. Literature data indicate [37] that MDH catalyses the pyridine nucleotide-dependent conversion of malate and oxaloacetate in the tricarboxylic acid cycle. It is also believed to play a key role in the transfer of malate/aspartate across the inner mitochondrial membrane. As Sekova et al. [38] reported, MDH plays an important role in adapting cells to stressful conditions in *Yarrovia lipolityca* yeast. The level of this protein increases under alkaline stress (pH 8.5). The results presented are consistent with our data, where selenium increased the abundance of this protein. In the case of ATP-dependent RNA helicase dbp3, they constitute a large family of proteins involved in the processes of RNA synthesis and degradation. The environmental stress caused by selenium has a great influence on the molecular processes taking place in the cellular cytosol. The abundance of this protein may have been the result of intensified processes involved in efficient pre-rRNA processing in yeast cells. The AlaX protein was also found to be abundant after yeast cultivation in the presence of 20 mg Se^4+^/L. The appropriate level of AlaX editing results in the correct incorporation of Ser into the Ala position in the proteome, which does not lead to a cascade of misfolding events or the degradation of key regulatory proteins. This is critical in protecting yeast cells that are exposed to stressful conditions. According to Kavoor et al. [34], the AlaX enzyme derived from *E. coli* is resistant to oxidative damage, providing an important stress resistance mechanism. Loss of function due to a deficiency in AlaX editing may destabilise the proteome and disrupt key cellular pathways, which may be fatal to yeast [39]. The information on the functions of the individual pathways described above seems to coordinate with each other when the cell is under the influence of selenium stress. In the presence of this element, yeast tries to optimise the expression level of various genes involved in the biosynthesis or import of, for example, amino acids (tryptophan). The genes responsible for fatty acid metabolism can also be upregulated to provide adequate protection and tolerance to yeast cells due to the effect of selenium. This means that fatty acid biosynthesis can help tolerate selenium stress, for example, by strengthening the cell membrane. Thus, it seems that tryptophan metabolism may play a key role in coordinating fatty acid biosynthesis [40]. However, the relationship between tryptophan and fatty acid synthesis is not yet understood in yeast cells. It should be noted, however, that the functioning of any metabolic pathway depends on the concentration of selenium and the time of yeast cell culture.

Studies presented by El-Bayoumy et al. [41] also showed protein expression as a result of the selenium enrichment of the yeast *Saccharomyces cerevisiae* (SelenoExcell^®^). It is worth noting that the concentration of selenium used to produce yeast rich in this element had no effect of the viability of yeast cells and the rate of biomass growth. The authors found that the selected proteins expressed by selenium were, among others: HSP 70 (heat shock proteins 70), elongation factor 2, pyruvate kinase, triosphosphate isomerase, and eukaryotic translation initiation factor 5A.

It should be emphasised that yeast has developed appropriate detoxification mechanisms that are able to neutralise the toxic effects of various elements present in the culture medium. The occurrence of stressful conditions activates the yeast cells’ defence mechanisms, which seek to detoxify selenium. According to the mechanisms described in the literature, the excess of an element present in the yeast cell cytosol is transported to the extracellular environment by membrane vesicles, or it can be accumulated in the vacuole [23,42,43]. It is worth noting that the adaptation of yeast to unfavourable breeding conditions occurs as a result of evolutionary changes and also as a result of specialised breeding work on yeast cells. It is noted in the scientific literature that the toxicity of selenium to microorganisms may be the reason for the similarity of the element to sulphur. It has been suggested that the non-specific incorporation of the amino acids SeMet and SeCys into proteins, in place of methionine and cysteine, may affect the growth of microorganisms in a selenium-enriched environment [6]. The element also interacts with glutathione (GSH), thus inhibiting the growth of yeast cells. The appropriate presence of GSH molecules in yeast may condition efficient selenium binding. It is suggested that the bioavailability of this element tends to increase then. Additionally, the hydrogen selenide produced, as one of the leading metabolites, is involved in the synthesis of all chemical forms of selenium that occur in the cells of microorganisms [6]. It should be noted, however, that according to the literature data, with a sufficiently large dose of selenium in the substrate, there is a greater probability of oxidative stress [44,45].

Increasingly, in recent years, there has been a tendency to introduce new raw materials for the production of yeast cell biomass, which can be used as a dietary supplement. The consequence of these changes is the emergence of new stress factors related to the accumulation of substances in yeast cell biomass, the appropriate concentration of which may be toxic. The presence of selenium in the culture medium may create stressful conditions for yeast cells. That is why it is so important to skilfully monitor and cultivate microorganisms in order to check the functioning of individual metabolic pathways. The results obtained are necessary to determine the proper functioning of a given organism under stressful conditions (in the presence of selenium).

Extending the scope of the proteome analysis on the yeast’s response to selenium will, in the future, make it possible to conduct a comprehensive analysis of the sensitivity of cells to unfavourable factors, which may result from many criteria that are set for yeast cells, which are widely used in many industrial branches. Proteomics research will revolutionise scientists’ view of the role of proteins in controlling many processes occurring in yeast cells under stressful conditions.

## 4 Conclusion

The presence of selenium in the culture medium may disrupt the functioning of intracellular enzymes, which in turn leads to disturbances in the metabolism of yeast cells. Increasing the knowledge about the functioning of adaptation mechanisms to specific stress conditions will hopefully minimise their negative impact on yeast cells. Properly selected breeding conditions and further laboratory work may contribute to increasing the yield of metabolites produced by yeast cells, an example of which may be selenium bioplexes. To understand the genetic basis of the cellular functions involved in the tolerance of yeast to selenium stress, future research should be carried out in the form of transcriptome analysis to differentiate expression profiles of yeast samples with/without selenium treatment.
